# White Blood Cell Segmentation by Color-Space-Based K-Means Clustering

**DOI:** 10.3390/s140916128

**Published:** 2014-09-01

**Authors:** Congcong Zhang, Xiaoyan Xiao, Xiaomei Li, Ying-Jie Chen, Wu Zhen, Jun Chang, Chengyun Zheng, Zhi Liu

**Affiliations:** 1 School of Information Science and Engineering, Shandong University, Jinan 250100, China; E-Mails: zhangcongcong@mail.sdu.edu.cn (C.Zha.); changjun@sdu.edu.cn (J.C.); 2 Department of nephrology, Qilu Hospital of Shandong University, Jinan 250012, China; E-Mail: xiaoyanxiao2007@163.com; 3 Department of Oncology, the Second Hospital of Shandong University, Jinan 250100, China; E-Mails: sdulixiaomei@163.com (X.L.); lz505@163.com (Y.-J.C.); qinghuanjn@gmail.com (W.Z.); 4 Department of Hematology, the Second Hospital of Shandong University, Jinan 250100, China

**Keywords:** white blood cell, segmentation, color space decomposition, k-means clusters

## Abstract

White blood cell (WBC) segmentation, which is important for cytometry, is a challenging issue because of the morphological diversity of WBCs and the complex and uncertain background of blood smear images. This paper proposes a novel method for the nucleus and cytoplasm segmentation of WBCs for cytometry. A color adjustment step was also introduced before segmentation. Color space decomposition and k-means clustering were combined for segmentation. A database including 300 microscopic blood smear images were used to evaluate the performance of our method. The proposed segmentation method achieves 95.7% and 91.3% overall accuracy for nucleus segmentation and cytoplasm segmentation, respectively. Experimental results demonstrate that the proposed method can segment WBCs effectively with high accuracy.

## 1. Introduction

The immune system, which is the third line of defense of the human body, protects the body from viruses, bacteria, and pathogens. This natural defense identifies and eliminates abnormal cells, such as tumor cells. The immune system consists of immune organs, immune cells, and immune molecules. White blood cells (WBCs) are the principal components of immune cells and play an important role in our body's immunity.

In fact, WBCs normally have a constant concentration in the human blood. If the amount of WBC exceeds the normal range, then health problems may occur. The morphological analysis of WBCs is one of the basic steps of blood pathological analysis. Morphological analysis is traditionally performed manually, thus making this a tedious and time consuming process, even for an expert. Furthermore, morphological analysis is limited to the professional knowledge and eyesight of a pathologist. Automatic methods have been developed in recent years to overcome the limitations of manual methods, which can cause calculation inaccuracies. Automatic devices use the principle of the light scattering method to calculate red blood cells (RBCs) and WBCs [1]. The counting procedure of the light scattering method is invisible and can only achieve routine classification, merely counting the number of RBCs and WBCs. Actually, WBCs can be divided into five types, namely, basophils, eosinophils, neutrophils, lymphocytes, and monocytes [2,3]. The first three types are granular types, and the last two types are non-granular. Distinguishing these types by using the light scattering method proved unsuccessful in this study. Thus, we used image analysis, which can provide human-like assessments, to overcome this problem. Image analysis identifies abnormal cells and adopts the diagnosis experience summarized by pathologists. This method uses the high resolution and flexible extracted features of computer vision to improve work efficiency and accuracy. Researchers are currently focused on developing a system that can automatically identify WBCs by using blood images. However, accurate WBC segmentation before classification still poses challenges.

WBC segmentation is an important part of the WBC classification system, and the segmentation results directly affect the accuracy of cell recognition. Researchers have proposed several useful methods for achieving accurate segmentation results. Huang *et al.* [4–7] divided WBC segmentation into two parts: one for nucleus segmentation and the other for cytoplasm segmentation. The nuclei of WBCs are the areas of high contrast and are relatively easy to segment in blood smear image. Thus, most of the traditional methods [4–7] first segment the nucleus part of the image before the cytoplasm part.

Considerable research has been done in this field in recent years. All these methods can be divided into five categories: threshold-based methods, learning-based methods, active-contour-based methods, metaheuristic-based methods, and saliency-based methods [8]. Threshold-based methods include the region growing method, watershed method, Otsu's method, and their combination. Duan *et al.* [6] segmented the nucleus and then applied the region growth of color information according to certain rules to extract the cytoplasm. Putzu *et al.* [9] proposed a method based on the thresholding of the cyan, magenta, yellow, key plate (CMYK) color space. Threshold-based methods have high running speed and reliable performance for unified images. However, the performance of threshold-based methods is inconsistent for non-unified images. Learning-based methods include supervised methods, including support vector machine (SVM), artificial neural network, and unsupervised methods such as k-means clustering and Fuzzy c-means. Zheng *et al.* combined the expectation-maximization clustering and SVM to segment the WBCs [10]. The performance of learning-based methods mainly depends on the selection of color feature vectors. Active-contour-based methods include level sets and snakes. Zamani *et al.* [7] segmented the nucleus first, applied a gradient vector flow (GVF) snake to segment WBCs, and used the boundary of the nucleus as the initial contour of the snake. Ko *et al.* [11] proposed a WBC segmentation method by using stepwise merging rules based on mean-shift clustering and boundary removal rules with GVF snake. Sadeghian *et al.* [12] presented a WBC segmentation framework that segments the cytoplasm by using GVF snake. This framework identifies the cytoplasm by applying Zack thresholding [13] to a gray image with the nucleus region removed. Active-contour-based methods are limited to the selection of initial contour, and overlapping objects will lead to poor performance of the methods. Metaheuristic-based methods include genetic algorithm (GA) and differential evolution, among others. Osowski *et al.* [14] presented a method that combines SVM and GA to recognize blood cells and used GA to select the features used by SVM. Metaheuristic-based methods regard segmentation problem as optimization problem, but the application of metaheuristic-based methods in WBC segmentation is very limited [8]. Saliency-based methods are based on visual saliency attention. Zheng *et al.* localized and segmented WBCs based on visual saliency attention [15]. Pan *et al.* realized WBC segmentation based on simulated visual attention, and gained satisfactory results [16]. Saliency-based methods are effective in the segmentation of WBCs. For some particular and practical applications, however, there is still some work to do. Some methods combine more than one of the categories listed above and achieve better results, such as combination of learning-based method and visual-attention-based method [17] and combination of learning-based method and thresholding-based method [18]. Other segmentation methods for WBC aside from the above five categories are available [19,20].

Saraswat *et al.* surveyed the current situation of WBC segmentation and indicated that the results of WBC segmentation are still not acceptable and need to be improved. They pointed out that the presence of artifacts, shape variations in the WBCs, and overlapped cells are the major problems and must be focused on [8]. Thus, aiming at the first two major problems, we present a novel, effective technique with good performance and robustness for WBC segmentation.

## 2. Technology Background

### 2.1. Color Transfer

Blood smear images are sampled with microscopic imaging systems. Given the different lighting conditions, varied dyeing time, unstable smear thickness, and different physical qualities of subjects, the color of blood smear image is changed in practice. Most of the published methods only work in databases constructed under strictly controlled conditions. By contrast, in practical application, the blood images to be detected do not always have good color consistency. Therefore, color adjustment for blood smear images is needed. This paper proposed a color adjustment method based on color transfer and k-means clustering, and this method will be discussed in the following sections. The color transfer method will also be introduced.

In 2001, Shirley *et al.* proposed the color transfer method between images [21]. This method is a general color correction method. The main idea of the method is to bring the color characters of one image to another image according to their mean value and standard deviation in Lab color model. In this paper, a standard template image that can be segmented accurately is chosen. The remaining images were then transformed according to the color characters of the template image. The algorithm can be elaborated into the following steps:

1. Transform the RGB color space of the input image and the template image to Lab color space.
2. Compute the mean value and standard deviation of the input image and the template image in Lab color space.
3. Subtract the mean value from all the pixels according to the following equation:
  (1) $$\{\begin{array}{c} l^{\prime }=l-\bar{l}\\ a^{\prime }=a-\bar{a}\\ b^{\prime }=b-\bar{b} \end{array},$$
  where *l*, *a*, *b* denote the L, a, b components of each pixel; *l̄*, ā, *b̄* denote the mean value of the L, a, b components; *l*′, *a*′, *b*′ denote the resultant value of each pixel after subtraction.
4. Scale the pixel points of each color component of the synthetic image by factors determined by the ratio of respective standard deviations:
  (2) $$\{\begin{array}{c} l^{\prime \prime }=\frac{\sigma_{t}^{l}}{\sigma_{s}^{l}}l^{\prime }\\ a^{\prime \prime }=\frac{\sigma_{t}^{a}}{\sigma_{s}^{a}}a^{\prime }\\ b^{\prime \prime }=\frac{\sigma_{t}^{b}}{\sigma_{s}^{b}}b^{\prime } \end{array},$$
  where $\sigma_{t}^{l}$, $\sigma_{t}^{a}$, $\sigma_{t}^{b}$ denote the standard deviation of the L, a, b components of the template image; $\sigma_{s}^{l}$, $\sigma_{s}^{a}$, $\sigma_{s}^{b}$ denote the standard deviation of the L, a, b components of the input image; *l*″, *a*″, *b*″ denote the resultant value of each pixel.
5. Transform the Lab color space of the resultant image to RGB color space.

**Figure 1.**
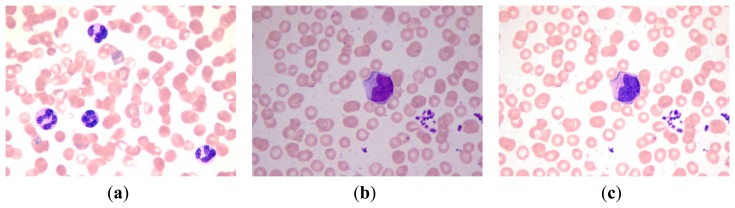
(**a**) Template image; (**b**) Input image; (**c**) Synthetic image.

Through the steps above, the color characters of the template image are transferred to the input image, and the synthetic image possesses the color features that are beneficial to WBC segmentation. The template image, the input image and the synthetic image based on the color transfer method are shown in Figure 1.

### 2.2. Different Color Spaces

The purpose of a color space (also called color model or color system) is to facilitate the specification of colors in some standard [22]. A color space is represented by matrix in mathematics, and typically, the matrix consists of three or four dimensions (e.g., RGB, HSI, and CMYK). The RGB color space is the most common color space used in electronic devices, such as cell phones and monitors. In the RGB color space, each color can be acquired by the addition of three primary colors: red, green, and blue. Therefore, this color space is an additive color space. The original stained blood smear image is represented by the RGB color space in the RGB model.

**Figure 2.**
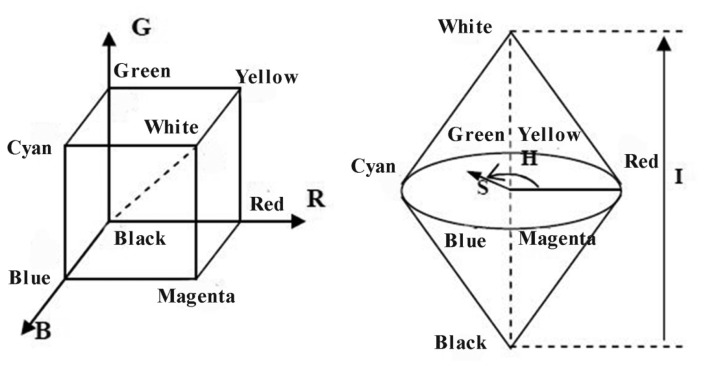
Schematic of the RGB color model (**Left**) and HSI color model (**Right**).

The HSI color space reflects the human vision system, where H denotes hue, S denotes saturation, and I denotes intensity. The I component is independent of color information, and the H and S components are closely related to the way humans perceive color. These characteristics make the HSI color space suitable for color detection and analysis. The schematic the of RGB and HSI color models is shown in Figure 2.

The CMYK color model is a subtractive color model and it is used in color printing. CMYK refers to the four inks used in color printing: cyan, magenta, yellow, and black. In the CMYK color space, C denotes cyan, M denotes magenta, Y denotes yellow, and K refers to the key plate (black).

The Lab color model is based on the way human perceive color, and it can describe all the color that human can see. This model minimizes the correlation between different color components. Color management system utilizes Lab as color patch to color transform one color space to another color space. Every color model has its own specific features, and the original stained blood smear image in the RGB color space shows useful features that benefit WBC segmentation when translated to HSI and CMYK color spaces. The details are discussed in the following section.

### 2.3. K-Means Clustering

K-means algorithm is a typical clustering algorithm based on distance. This algorithm adopts distance as the evaluation parameter of similarity, which means the shorter the distance is the more similar the two objects are. K-means clustering aims to partition *n* observations into *k* clusters. Each observation in k-means clustering belongs to the cluster with the nearest mean, which serves as the prototype of the cluster. We take advantage of this feature to partition M×N pixels in the stained blood smear image into several clusters on the basis of the color information of the background, RBCs, cytoplasm, and white cell nuclei. M denotes the number of rows in the image, and N denotes the number of columns in the image.

In the clustering problem presented in this paper, the training set consisted of each pixel in the image and was denoted as {*x*^(1)^, *x*^(2)^, …, *x*^(*L*)^}, where *x*^(*i*)^ ∈ ℝ*^n^*, i ∈ [1 L], L = M×N. Each sample *x*^(*i*)^ of the training set is a vector decided by the combination of color components of different color spaces. The adopted algorithm is described by the following steps:

(1) Select an initial set of k-cluster centroids μ_1_, μ_2_, …,μ*_k_* ∈ℝ*^n^* randomly.
(2) After assigning the initial k-cluster centroids, the algorithm proceeds by alternating between two steps until the steps converge:

**Assignment step:** For each sample *x*^(*i*)^, decide the cluster of a sample by calculating the squared Euclidean distance between sample *x*^(*i*)^ and each cluster centroid μ*_j_* (j ∈ [1 k]) according to Equation (3):

(3) $$c^{\left(i\right)}:=\arg\text{min}||x^{\left(i\right)}-\mu_{j}|{|}^{2},$$

where *c*^(*i*)^ denotes the cluster number of *x*^(*i*)^.

**Update step:** For each cluster, recalculate the new means to be the centroids of the observations in the new clusters according to Equation (4):

(4) $$\mu_{j}:=\frac{\sum_{L}^{i=1}1\{c^{\left(i\right)}=j\}x^{\left(i\right)}}{\sum_{L}^{i=1}1\{c^{\left(i\right)}=j\}}.$$

## 3. Proposed Method

**Figure 3.**
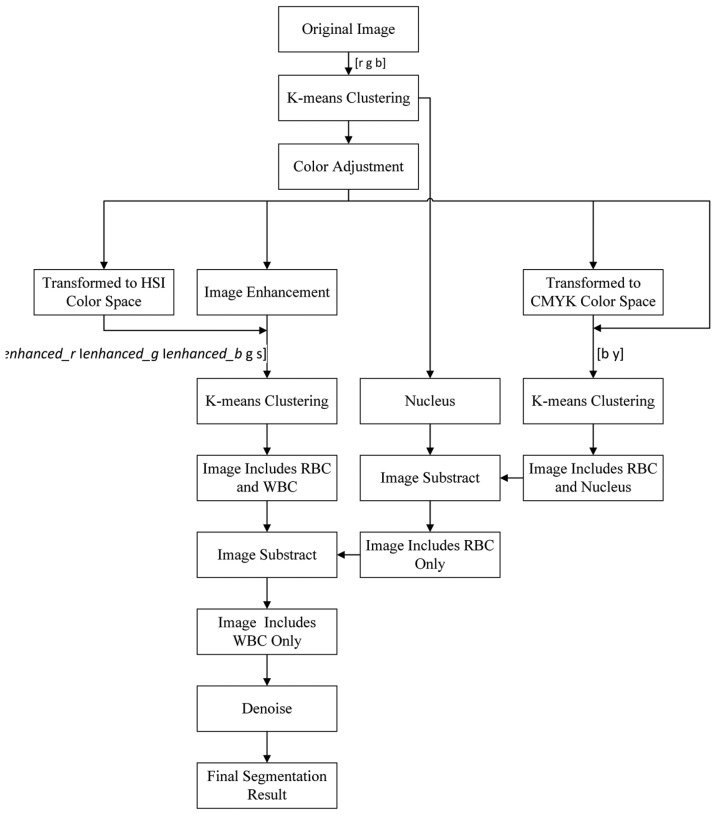
Flow chart of the proposed method.

We adopted k-means clustering three times on the basis of the color information in different color spaces to segment the nucleus and cytoplasm of WBCs to achieve good performance. The entire flow chart of the proposed method is presented in Figure 3.

### 3.1. Color Adjustment

In Section 2.1 a general color correction method was introduced. However, complicated backgrounds degrade the performance of the method. To achieve satisfactory results, a color adjustment method based on color transfer and k-means clustering is introduced. The main idea is to divide the blood smear image into several parts by using k-means clustering. The color transfer algorithm will be applied for each part of the image, and the synthetic image can be obtained by reunion of the parts.

The stained blood smear images can be divided into three parts based on the following information: the background, the RBCs, and the WBC nuclei. Each pixel in each part has specific features. K-means clustering is used to segment the background, RBC, and the nucleus.

**Figure 4.**
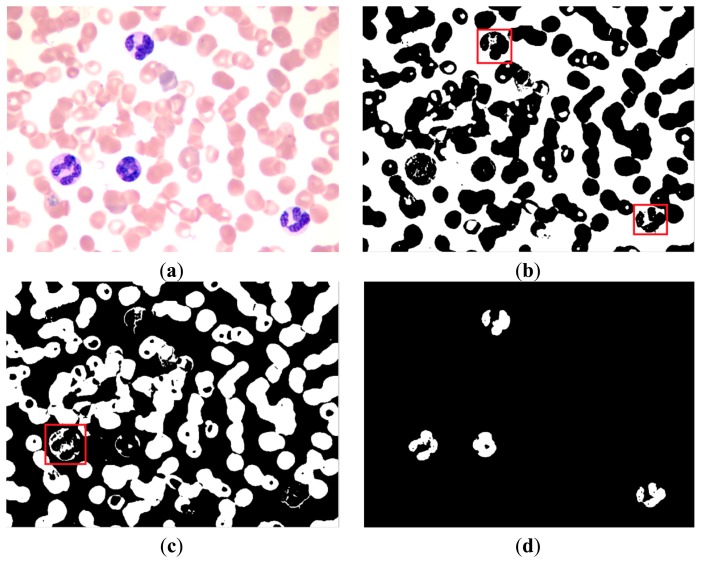
(**a**) Original image; (**b**–**d**) Partition results after clustering.

The original image in the RGB color space was a M × N × 3 matrix. The value of each pixel was a 1 × 3 vector such as [r g b], where r denotes the R component value of this pixel, g denotes the G component value, and b denotes the B component value. The [r g b] of each pixel was treated as a feature vector; the original M × N × 3 matrix was reshaped to an L × 3 matrix (L = M × N), then k-means clustering was used to cluster every pixel into three partitions. Figure 4 shows the partition results.

**Figure 5.**
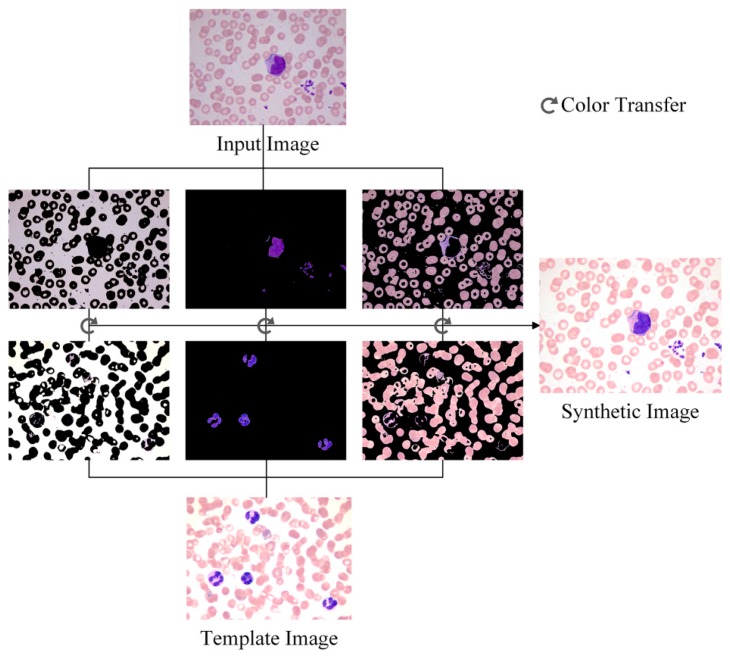
Schematic plot of the proposed color adjustment method.

After clustering, the template image and the input image are divided into three parts respectively. As shown in Figure 5, the color transfer algorithm is applied to each part of the template image and the input image. The synthetic image can be obtained by the reunion of the three parts, and the subsequent steps are based on the synthetic image.

### 3.2. Red Blood Cell and Nucleus Segmentation

In Figure 4b, the white area represents the background of the image and contains part of the WBC cytoplasm because of the similar colors, as shown in the red block of the image. In Figure 4c, the white area represents the RBC region and part of the WBC cytoplasm, as shown in the red block of the image. The white area of Figure 4d represents the WBC nucleus region, and the segmentation result is relatively high because of high contrast.

We could not segment the cytoplasm of WBCs or RBCs directly from these results. Figure 4b shows that the white regions not only consists mostly of background but also contains the cytoplasm region; thus, removing the background of the image is unfeasible. Figure 4c shows that most of the white parts are RBCs but also contains the cytoplasm; thus, the cytoplasm region was difficult to remove and was deemed unsuitable to segment RBCs.

**Figure 6.**
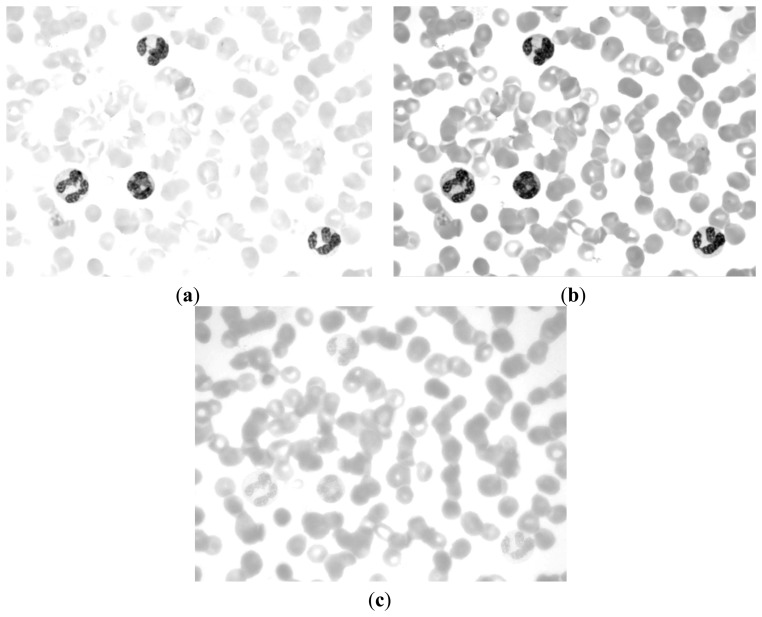
(**a**), (**b**) and (**c**) R, G, and B components of the original image respectively.

**Figure 7.**
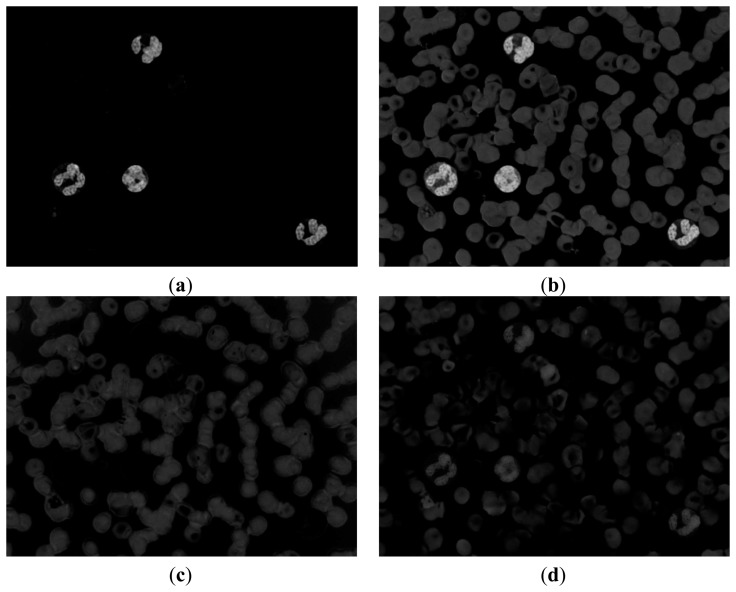
(**a**), (**b**), (**c**) and (**d**) C, M, Y, and K components of CMYK color space image respectively.

If we can determine a way to segment the RBC and nucleus together, then we can obtain the RBC region by subtracting the nucleus region obtained above. The R, G, and B components of the original image are shown in Figure 6. The CMYK color space image is translated from the RGB color space. The C, M, Y, and K components of the CMYK color space image are shown in Figure 7.

**Figure 8.**
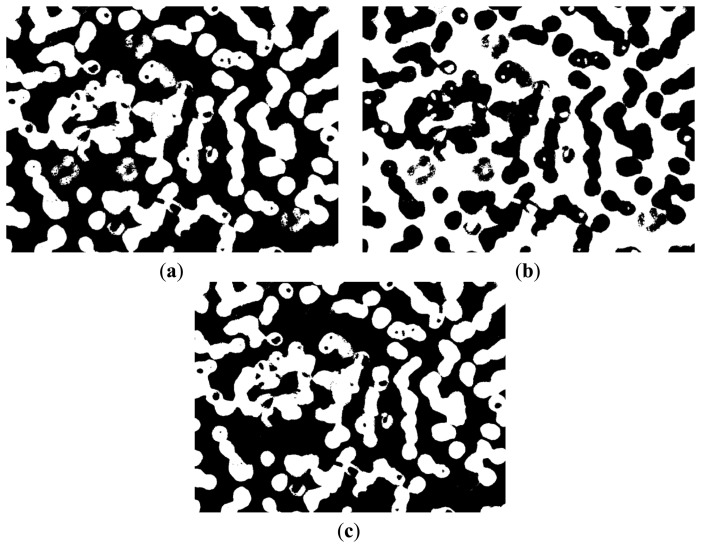
(**a**), (**b**) Clustering results; (**c**) RBC segmentation results.

Figures 6c and 7c demonstrate that the RBC and nucleus regions have more contrasts than the cytoplasm in B and Y components. We can use this result to segment the RBCs and nuclei together. By combining B and Y components into a M × N × 2 matrix, the value of each pixel will be a 1 × 2 vector denoted as [b y], where b denotes the B component's value of this pixel and y denotes the Y component's value of this pixel. The [b y] of each pixel was considered a feature vector; the original M × N × 2 matrix was reshaped to an L × 2 matrix (L = M × N); and k-means clustering was adopted to cluster every pixel into two partitions. The results after partition are shown in Figure 8.

Figure 8a shows that this image contains RBC and nucleus regions only. The RBC region could be obtained by using Equation (5), and the result is shown in Figure 8c. The segmented RBCs may appear smaller than the actual cells; thus, morphological dilation was applied to expand the size of the RBC region to approximate the real one.

(5) $$I_{\text{RBC}}=I_{\text{RBC}\_\text{Nucleus}}-I_{\text{Nucleus}\;},$$

where *I_RBC_* denotes the binary image that contains RBCs only; *I_RBC_Nucleus_* denotes the binary image obtained above that contains RBC and nucleus (Figure 8a); *I_Nucleus_* denotes the binary image that contains the nucleus only (Figure 4d).

### 3.3. Cytoplasm Segmentation

**Figure 9.**
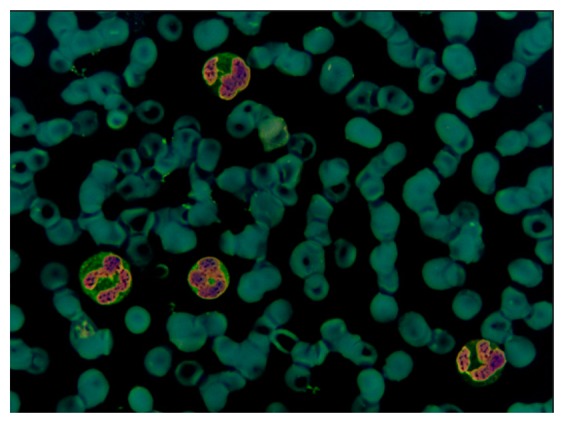
Enhanced image.

Cytoplasm segmentation is the most difficult part of WBC segmentation because the color of a cytoplasm is usually similar to the color of the RBCs or the background. The original image first needs to be enhanced to segment the cytoplasm. To enlarge the contrast of each content, the following equation was applied to every component of the original image:

(6) $$I_{\text{enhanced}}=I+I_{\text{bothat}}-I_{\text{tophat}},$$

where *I_enchanced_* denotes the enhanced RGB color space image, I denotes the original image, *I_bothat_* denotes the image after bottom hat transformation, and *I_tophat_* denotes the image after top hat transformation. Figure 9 shows that the cytoplasm region has high contrast in the enhanced image.

**Figure 10.**
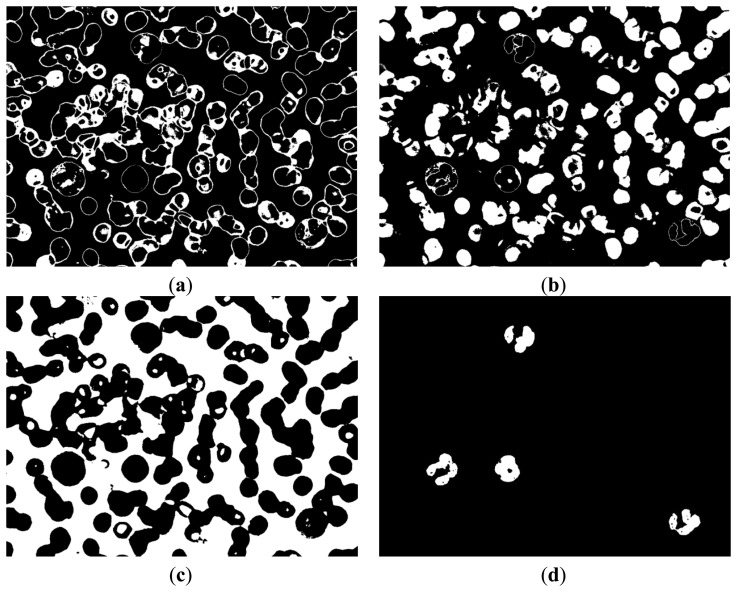
Clustering results of *I_enchanced_*.

Figure 10c shows that the segmentation results of the background are better than the results in Figure 4b. Furthermore, the segmentation results of the background do not contain the cytoplasm region. Most of the cytoplasm parts are contained in Figure 10a, and some are contained in Figure 10b.

**Figure 11.**
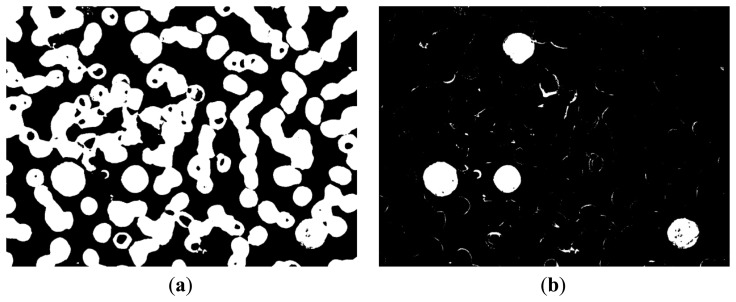
(**a**) Result of the whole segmentation of the WBC and RBC regions; (**b**) Preliminary result of the entire WBC region segmentation.

**Figure 12.**
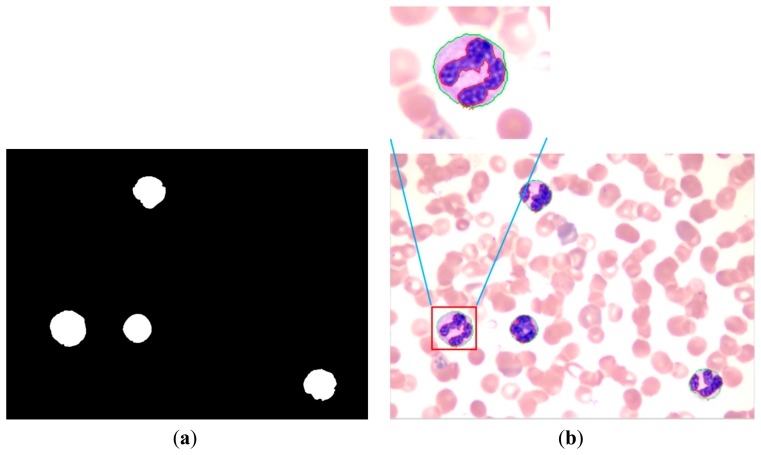
(**a**) WBC segmentation result after denoising; (**b**) Result of WBC nucleus and cytoplasm segmentation.

The entire WBC and RBC regions were obtained (Figure 11a) by combining the results of Figure 10a,b. The RBC region was also obtained (Figure 8c), and the WBC region could be acquired by subtracting Figure 8c from Figure 11a, with the result shown in Figure 11b. Figure 11b shows that the influence of noise on the WBC segmentation results. Thus, denoising is a necessary step. Smaller noises are prominent in the preliminary result of the entire WBC region segmentation; this result can be used to remove noises. First, an open operation was conducted to remove small dots and to separate WBC from small noises that connect to it. Second, all connected components were labeled and their sizes were computed before removing connected components with sizes smaller than the threshold value. After removing the noises outside the WBC, subsequent steps are needed to ensure the completeness of WBC. Third, the holes in WBC were filled and narrow gaps were connected by close operation. The WBC segmentation result after denoising is shown in Figure 12a. The cytoplasm part could be obtained by subtracting the nucleus region from the entire WBC region. The WBC nucleus and cytoplasm segmentation results are shown in Figure 12b.

## 4. Experimental Results

**Figure 13.**
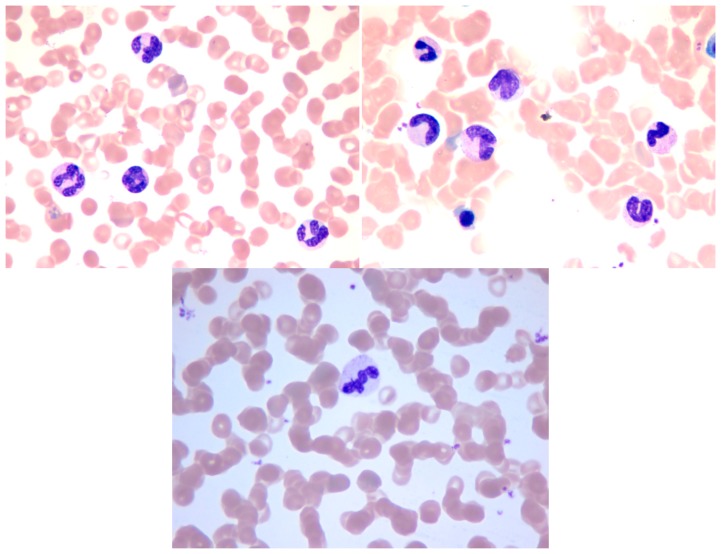
Sample images in the database.

Several methods for WBC segmentation have been proposed. However, the majority of these methods work only in specific databases wherein images are captured under certain circumstances to control light and other influencing factors. Our database was constructed by using an Olympus BX51 microscope in the cell lab at the Second Hospital of Shandong University under different circumstances. Our database consisted of 300 stained blood smear images. The images of the database were captured in three sections, and relatively long time gaps exist between each section. Approximately 100 images were obtained in each section of WBC image acquisition. In each acquisition section, the WBC images were captured under the same light condition from different blood smears. Between each acquisition section the light condition differs from each other to some extent. The blood smears used for acquisition were stained with the same dyeing technique, and the dyeing time and the smear thick of each smear was slightly different according to the experience of the hematologist. Every WBC image has at least one WBC, and a number of them may have several WBCs. As previously mentioned, five types of WBCs can be found in the database. Every WBC image contains at least one type of WBC, and a number of them may contain several types of WBCs. The database has a total of 690 WBCs, which consists of 87 basophils, 94 eosinophils, 124 neutrophils, 203 lymphocytes, and 182 monocytes. Some sample images in our database are shown in Figure 13. The samples show that the color of each image differs from one another.

Different databases differ in background color and dyeing conditions. Thus, not all of the previously proposed segmentation methods mentioned in the Introduction perform well all the time. The different conditions of the blood smear image hinder WBC segmentation. By conducting various experiments, we developed a method to segment WBC. Our method consists of two parts: (1) the segmentation of the nucleus and RBC; and (2) the segmentation of the WBC cytoplasm. By acquiring the whole WBC region and the nucleus region, the cytoplasm region could be obtained by a simple subtraction operation. During processing, we assumed that every input image had at least one WBC; no inferences were sizable compared with the WBC contained in the image. We also ignored the overlapping WBCs issues.

We present the performance test of the proposed WBC segmentation method on our database in this section, as discussed in Section 3. A total of 300 blood smear images were used to test the performance. We applied our method on each of the 300 images and then compared the results with the results of manual segmentation. The percentages of accuracy were calculated accordingly. Our database obtained an average overall accuracy of 95.7% for nucleus segmentation and 91.3% for cytoplasm segmentation. In this evaluation, the segmentation result is considered accurate when the auto-detected boundary closely matches the manually traced boundary.

We compared our method with the region growing method [6] and the snake method [8] to further evaluate the performance of our method. Table 1 shows the experimental results, wherein A1 denotes the ratio of the number of correctly detected WBCs to the total number of detected WBCs (Equation (7)). When A1 = 1, the WBCs detected in the microscopic images are all real WBCs, and no false detection occurs. The evaluation parameter A2 denotes the ratio of the number of WBCs detected to the total number of WBCs, as shown by Equation (8). When A2 = 1, no leak detection occurs. Equation (9) demonstrates the computation of false and leak detection ratios. A1 and A2 denote the detection accuracy of the entire system. In evaluating A1 and A2, we disregarded the accuracy of the traced boundaries. Therefore, some errors are allowed in a certain margin between the auto detected and manually traced boundaries; that is, the detection focuses only on the rough relative position between the traced boundaries and real WBCs. Therefore, the results are better than the results in the overall evaluation. D denotes the average Hausdorff distance between the auto detected and manually traced boundaries. Several experts are requested to manually trace the boundaries of WBC. We treated the average result of the experts as the ground truth and then applied the Hausdorff distance between the auto detected and manually traced boundaries to describe their contact ratios. A smaller D corresponds to better performance. D has a close relationship with the recognition accuracy of the entire system because the features extracted from the detected WBCs strongly rely on the segmentation results, whereas D describes the segmentation performance:

(7) $$\text{A}1=\frac{\text{The number of correctly detected WBCs}}{\text{Total number of detected WBCs}}\times 100\%,$$

(8) $$\text{A}2=\frac{\text{Total number of detected WBCs}}{\text{Total number of WBCs that exist in all images}}\times 100\%,$$

(9) $$\{\begin{array}{c} E1=1-\text{A}1\\ E2=1-\text{A}2 \end{array}\;,$$

where E1 denotes the false detection ratio and E2 denotes the leak detection ratio.

**Figure 14.**
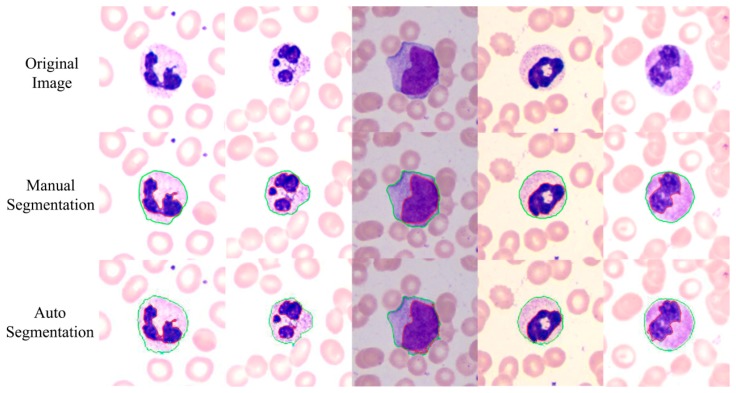
Comparison of the proposed method and the manual method on single WBC segmentation.

**Figure 15.**
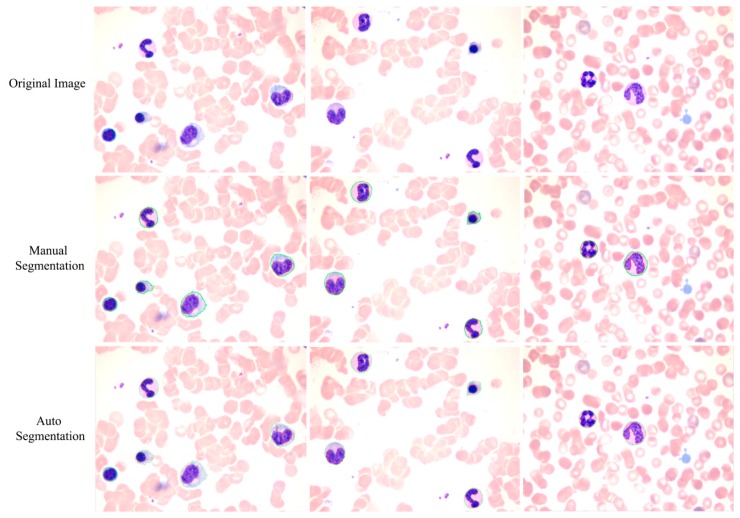
Comparison of the proposed method and the manual method on multiple WBC segmentation.

The proposed method achieves a high overall accuracy for cytoplasm segmentation. Table 1 shows that the proposed method obtains low false and leak detection ratios and that the traced boundaries of the proposed method achieves high accuracy. The segmentation results for single WBC of the proposed method are compared with the original images and the manual segmentation results in Figure 14. The proposed method also performs well when multiple WBCs exists in one image, as shown in Figure 15.

The performance of the proposed method, region growing method, watershed method and snakes' method was also evaluated via the parameter: OR, UR, ER and RDE [16,23,24]. OR denotes the over-segmentation rate, UR denotes the under-segmentation rate, and ER denotes the overall error rate. They are often used to evaluate the performance of segmentation methods. Pan *et al.* [16] and Liu *et al.* [23] have given a clear definition. RDE denotes the relative distance error, which is firstly proposed by Yang-Mao *et al.* [24] to evaluate the segmentation results. Assume that *e*_1_, *e*_2_, *e*_3_, …, *e_n_e__* are the pixels of E, and *t*_1_, *t*_2_, *t*_3_, …, *t_n_t__* are the pixels of T, in which E denotes the segmentation results, T denotes the ground truth, and *n_e_*, *n_t_* denote the number of pixels of E and T, respectively. The definition of RDE is illustrated as follows:

(10) $$\text{RDE}=\frac{1}{2}\left(\sqrt{\frac{1}{n_{e}}\sum_{i=1}^{n_{e}}d_{e_{i}}^{2}}+\sqrt{\frac{1}{n_{t}}\sum_{j=1}^{n_{t}}d_{t_{j}}^{2}}\right),$$

where *d_e_i__* = *min*{*distance*(*e_i_*, *t_j_*)|*j* = 1,2,…*n_t_*}, *d_t_j__* = *min*{*distance*(*e_i_*, *t_j_*)|*i* = 1,2,…*n_e_*}, and *distance*(*e_i_*, *t_j_*) denotes the Euclidean distance between *e_i_* and *t_j_* [16].

Table 2 shows the evaluation of the four methods via the average error measure of OR, UR, ER and RDE. The proposed method is only slightly worse than the region growing method in UR evaluation. It shows similar attributes to the watershed method in OR evaluation, however it is better in other indexes. Furthermore, the proposed method achieves a higher overall error rate of 14.3% and a lower relative distance error of 1.52 than the other compared methods. The evaluation demonstrates that the proposed method generally outperforms the traditional methods.

The performance of the proposed method on each type of WBC was evaluated. Table 2 shows the segmentation accuracy for each type of WBC. The performance of five kinds of segmentation methods was also compared. For each kind of method, a typical algorithm mentioned in the Introduction was evaluated as a representation. Duan's work [6] was adopted for the threshold-based method; Zheng's work [9] was adopted for the learning-based method; Zamani's work was adopted [7] for the active-contour-based method; Stanislaw's work [13] was adopted for the metaheuristic-based method; Zheng's work [14] was adopted for the saliency-based method. All of these methods were compared with the proposed method in Table 3, and the results show the proposed method achieved higher segmentation accuracy than those other methods.

In addition, owing to the color variety of blood smear images some segmentation methods that are sensitive to color space may fail to correctly segment WBCs. Most of the WBC segmentation methods focus mainly on the segmentation part, but few of them pay attention to color adjustment. However, the blood smear images captured in the clinic do not always have good color consistency. Thus, color adjustment is a necessary step in practical application. A color adjustment method based on k-means clustering and color transfer was adopted in this paper. The color adjustment was treated equally as the segmentation part. Experiments show that the color adjustment can improve the accuracy of WBC segmentation. Table 4 shows the difference between the proposed method with color adjustment and without color adjustment.

## 5. Conclusions

This paper proposed a new WBC segmentation method by using color-space-based k-means clustering. A novel color adjustment method was applied before segmentation, thus improving the segmentation accuracy. The color components of RGB, HSI, and CMYK color spaces were applied to form the feature vectors of the k-means cluster. The k-means clustering results provide information that can be used for WBC segmentation. The experimental results show that the proposed method achieves precision rates of 94.6% and 95.1% for evaluation parameters A1 and A2, respectively, and higher segmentation accuracy for each type of WBCs. The experimental results indicate that the proposed method achieves high overall accuracy in cytoplasm segmentation and small leak and false detection ratios. The traced boundaries of the proposed method are more accurate than those of other methods. Moreover, the proposed method is immune to light conditions to some extent and is robust. All of the abovementioned characteristics demonstrate that the proposed method is accurate and effective for WBC segmentation, and outperforms traditional methods.

## Figures and Tables

**Table 1.** Performance evaluation.

**Parameter**	**Region Growing**	**Snake**	**Proposed Method**
A1	88.4%	90.3%	94.6%
A2	89.2%	87.4%	95.1%
D	60.62	30.62	19.13

**Table 2.** Performance evaluation via average error measure of OR, UR, ER and RDE.

**Parameter**	**Region Growing**	**Snake**	**Watershed**	**Proposed Method**
OR	0.093	0.084	0.066	0.063
UR	0.059	0.081	0.068	0.067
ER	0.232	0.195	0.186	0.143
RDE	9.34	6.46	4.54	1.52

**Table 3.** Performance evaluation of each types of WBC separately.

**Methods**	**Neutrophil**	**Lymphocyte**	**Monocyte**	**Eosinophil**	**Basophil**	**Average**
Threshold-based method [6]	88.7%	90.1%	92.3%	87.2%	83.9%	89.3%
Learning-based method [9]	94.4%	95.6%	97.3%	87.2%	85.0%	93.3%
Active-contour-based method [7]	91.9%	94.6%	94.5%	88.3%	86.2%	92.2%
Metaheuristic-based method [13]	94.4%	93.6%	96.2	93.8%	88.5%	93.5%
Saliency-based method [14]	95.2%	98.1%	95.6%	94.5%	85.1%	94.1%
Proposed method	97.6%	97.0%	97.8%	89.4%	89.7%	95.7%

**Table 4.** Performance evaluation with and without color adjustment.

**Parameter**	**With Color Adjustment**	**Without Color Adjustment**
A1	94.6%	90.2%
A2	95.1%	89.4%
D	19.13	29.43
